# COVID-19 Impact On Black and Latina Women: Pregnancy and Parenting

**DOI:** 10.1007/s40615-024-02266-9

**Published:** 2025-01-13

**Authors:** Zoe Carrasco, Aliyah Behimino, Mariah Jiles, Brianne Taylor, Chakiya Clary, Gabriela Negrete, Andrea V. Aponte, Brittany D. Chambers Butcher, Anu Manchikanti Gomez, Stephanie Arteaga

**Affiliations:** 1 School of Nursing, University of California, 505 Parnassus Ave, San Francisco, CA 94143 USA; 2 Sexual Health and Reproductive Equity Program, School of Social Welfare, University of California, 110 Haviland Hall, MC 7400, Berkeley, CA 94720-7400 USA; 3 California Preterm Birth Initiative, University of California, 490 Illinois Street, Flr. 9 Box 2930, San Francisco, CA 94143 USA; 4 Department of Human Ecology, College of Agricultural and Environmental Sciences, One Shields Avenue, Davis, CA 95618 USA

**Keywords:** COVID-19; Maternal health, Parenting, Social determinants of health, Black women, Latina women

## Abstract

The coronavirus-19 (COVID-19) pandemic presented unique challenges for pregnant women and birthing individuals, particularly those from Black and Latino communities. Understanding the impact of the pandemic on their experiences is crucial for providing adequate support and care during vulnerable times. This research delves into the specific effects of COVID-19 on maternal stress and resilience. We conducted in-depth interviews with a subsample of 19 women from a larger study examining the effects of maternal stress and anxiety, racism, and resilience and coping on pregnancy among Black and Latina pregnant women in the San Francisco Bay Area, a diverse region where nearly two-thirds of residents are people of color. Using thematic analysis, we identified three dominant themes that illuminate the impact of the COVID-19 pandemic on participants’ pregnancy, birth, and postpartum experiences. Firstly, the pandemic shifted participants’ focus away from their pregnancy and birth experiences, compelling them to prioritize safety measures against COVID-19 infection. Secondly, the study highlighted the profound value of high-quality, supportive care from healthcare providers during the pandemic, which significantly impacted participants’ well-being. Lastly, we uncovered various resilience-building strategies employed by participants to navigate the challenges of pregnancy and parenting during the pandemic. This research provides essential insights into the lived experiences of Black and Latina pregnant women in the San Francisco Bay Area during the COVID-19 pandemic. The findings underscore the need for targeted support and interventions to address the unique stressors faced by these communities. By understanding the personal lived experience of Black and Latina participants’ pregnancy, birth, and postpartum during the pandemic, healthcare providers and policymakers can develop more tailored and effective approaches to assist and empower Black and Latina pregnant individuals.

## Introduction

Prior to the COVID-19 pandemic, in the USA, Black and Latino communities experienced disparate maternal health outcomes. Black women are three to four times more likely to die from pregnancy-related causes than non-Hispanic white women [1]. This devastating inequity has existed for more than 100 years and has widened over the last century, particularly in recent years [2]. Black women also experience significantly higher rates of preterm birth compared to women of every other ethnic and racial group; in 2018, the preterm birth rate for Black women was 14.1% compared to 9.1% for non-Hispanic white women, a rate that has been increasing in recent years [3]. These inequities in maternal mortality persist across income and educational levels; notably, Black women with a college-level education or higher are at increased risk of preterm birth and pregnancy-related mortality than white women with less than high school education [4, 5].

While Latina women in the USA might not share the same elevated risks for maternal and infant health deaths, they do experience higher rates of maternal morbidities, certain comorbidities during pregnancy, and depression during the perinatal period compared to the general population [6]. We acknowledge different terminology is used to describe people of Latin descent to be more inclusive of transgender and gender non-conforming people. When citing other data, we mirror the terminology used by the study authors to avoid giving the impression that these studies were inclusive of the experiences of the full spectrum of gender identity. For our study, we use the terms Latina and Latino to reflect the cultural norms of the countries (e.g., Mexico and Central America) of origin of our foreign-born population. A study of 2.2 million Blue Cross-insured hospital births from January to October 2020 examined severe maternal morbidity amongst Black, Hispanic, and white women. The outcome showed that, among their insured population, Black and Hispanic women had a drastically higher rate of severe maternal morbidity risk factors compared to white women (63% and 32% higher, respectively) [7]. There is also evidence that Hispanic women are at higher risk for gestational diabetes mellitus compared to non-Hispanic white individuals [8]. Access to culturally relevant care for Latino people is also a pertinent issue. A 2018 poll of 1522 U.S. Latino adults conducted by the Associated Press-NORC Center for Public Affairs Research found that nearly 6 in 10 respondents reported experiencing difficulty communicating with a healthcare provider due to language or cultural barriers [9].

COVID-19 had a profound impact on maternal health, affecting women and healthcare systems [10]. The pandemic led to disruptions in routine prenatal, labor, and postnatal care. The direct consequences of these healthcare modifications led to Black and Latino communities being disproportionately impacted. In 2022, the United States Government Accountability Office (U.S. GAO) found that between 2019 and 2021, the maternal death rate for non-Hispanic Black women rose from 44.0 to 68.9 per 100,000 live births, and for Latina women, it increased from 12.6 to 27.5 per 100,000 live births. The U.S. GAO highlighted a rise in preterm birth rates, with non-Hispanic Black and Hispanic women experiencing higher rates than their non-Hispanic white counterparts. Between 2020 and 2021, the preterm birth rate for non-Hispanic white women rose from 9.1 to 9.5%, while for Hispanic women, it increased from 9.8 to 10.2%. Non-Hispanic Black women had the highest preterm birth rate, rising from 14.4 to 14.8% during the same period.

The rapid reconstruction of the entire healthcare system during COVID-19 directly impacted maternal health, while the economic burden from shelter-in-place measures created an indirect impact [10]. While numerous employers have allowed their employees to work from home to reduce the risk of COVID-19 infection, many Black and Latino people did not benefit from this shift to virtual work. An analysis of the Current Population Survey examined employment patterns between May 2020 and July 2021 and found that Black and Hispanic workers had 35% and 55% lower odds of teleworking, respectively, compared to white workers [11]. Importantly, research shows that people of color, including those identifying as Black and Latino, were more likely to work in essential industries and frontline occupations in the year before the COVID-19 pandemic [12], and were more likely to experience a decline in employment shortly after the onset of the pandemic [13].

To mitigate the negative financial impact of the COVID-19 pandemic on Americans, the United States government began issuing Economic Impact Payments for eligible individuals in mid-2020 and provided tax credits to parents of children under the age of 18 [14]. However, there were disparities in receipt of these benefits. As of May 2020, nationally representative data of nonelderly adults showed that only 69% percent of non-Hispanic Black adults and 64% percent of Latino adults reported receiving the federal stimulus payment, compared to 74% percent of non-Hispanic white adults [15]. Further, only 59% of adults with incomes below or at the federal poverty line reported receiving the stimulus payment, compared to 78% of those with higher incomes. Only 54% percent of Latino adults in families with noncitizens reported receiving this payment [15].

There is limited research regarding the pregnancy, birth, and postpartum experiences during the height of the COVID-19 pandemic, but some studies show decreased levels of support during hospital births due to shifting policies and procedures in hospitals to curb the spread of COVID-19 [16]. Hospital policies throughout the country varied, with many hospitals only allowing one support person or even no support people at the height of the pandemic [17], despite research suggesting positive outcomes associated with continuous support during labor [18]. In one study of a convenience sample of 885 women aged 18 to 43 who gave birth in U.S. hospitals between March and July 2020, 61% of women reported that they received inadequate support for childbirth [19]. Furthermore, 33% of these women reported experiencing anxiety, 18% reported experiencing depression, and 20% of women reported not feeling safe giving birth in a hospital during the pandemic [19]. In this analysis, we explore the lived birthing and parenting experiences of presumed cisgender Black and Latina women before and after the onset of the COVID-19 pandemic in a diverse metropolitan city, the San Francisco Bay Area.

Our study aims to explore the pregnancy and parenting experience during COVID-19 from the voices of mothers in the San Francisco Bay Area. The San Francisco Bay Area is home to many Black and Latino families and experienced high incidence and mortality rates among these populations at the onset of the pandemic. We gathered patterns and data from the perspectives of cisgender Black and Latina pregnant and postpartum women that we hope to learn from in order to improve maternal outcomes. While we acknowledge that not all individuals giving birth in this population identify as women, our study sample focuses on individuals who do, thus, we use the term “women” when describing the participants.

## Methods

We utilized data from the Supporting Our Ladies and Reducing Stress to Prevent Preterm Birth (SOLARS) Study. SOLARS is a mixed method, longitudinal study examining the effects of maternal stress and anxiety, racism, and resilience and coping on pregnancy among Black and Latina pregnant women and birthing people in the San Francisco Bay Area. To be eligible for the study, individuals had to identify as Black or Latina, be 18–44 years of age, live or work in Oakland or San Francisco California, and be pregnant. More information on the SOLARS study can be found elsewhere (*SOLARS study*, n.d.). The present qualitative study examined the effects of the COVID-19 pandemic on stress during pregnancy among a subsample of the 77 SOLARS participants who successfully enrolled in the study.

The San Francisco Bay Area was chosen as the research location due to its racial and ethnic diversity. The Bay Area is a majority non-white region, with two-thirds of residents being people of color. The region has a large Latino population, with nearly a quarter of residents identifying as Latino as of 2020. While the region overall and the city of San Francisco comprise a relatively small proportion of Black residents at 6%, Alameda County, where the city of Oakland is located, has a larger proportion of Black residents than the region overall at 9%. Thus, the Bay Area’s diversity makes it a relevant region to assess the impacts of COVID-19 on the maternal health of Black and Latina women [20].

We recruited SOLARS participants to join the qualitative study utilizing purposive sampling to ensure the representation of participants who were Black and Latina, already parenting at the time of enrollment, and who gave birth prior to or after the onset of the COVID-19 pandemic. Authors ZC, SA, and BT conducted in-depth interviews between December 2020 and March 2021 using an open-ended guide designed to capture participants’ experiences during pregnancy and birth; maternal and infant healthcare visits; the COVID-19 pandemic; and current events related to immigration and racial justice (i.e., the reckoning of racialized violence by police after the death of George Floyd). Prior to engaging in data collection, members of the interviewing team new to qualitative interviewing participated in a training led by study primary investigators AMG and BCB. All interviewers conducted practice interviews before interviewing participants. All interviewers identified as women and Black or Latina and were matched to participants by gender and most (12/19) by race or ethnicity. To keep patients and interviewers safe during the pandemic we conducted the interviews via telephone or video conference. Interviews were conducted in participants’ preferred language (English or Spanish). Interviewers completed a memo after each interview to capture important takeaways. Participants received a $50 gift card incentive upon completion of the interview. Interviews lasted an average of 65 min and were audio recorded and transcribed. ZC and AB reviewed transcripts and audio to ensure the accuracy of transcription. The [IRB blinded] approved the study protocol.

ZC and AB, guided by SA, conducted a thematic analysis (TA) to analyze the data utilizing both codebook and reflexive thematic analysis approaches [21]. Codebook TA was beneficial to us in working with multiple coders/analysts and for exploring the predetermined topic of the impact of COVID-19 on pregnancy, birth, and parenting [21]. However, reflexive TA’s emphasis on critical reflection allowed us to examine our respective positionalities and their impact on analysis, particularly the interpretation of the data [22]. Throughout the analysis process, ZC and AB regularly met to discuss how their own personal experiences and values interacted with their interpretation of the data, and each wrote a memo to examine these experiences and values. For example, ZC, a midwifery student and Latina-identified woman, noticed how her experiences growing up in a Latino family were present in her orientation toward and interpretation of the data as she recognized similarities with her own family members in the study’s participants, while AB, a Public Health student and Filipina-identified woman, noticed how her friendships with women who have recently given birth influenced how she interpreted the pregnancy and postpartum experiences of study participants as she noticed similarities in the unique joys and challenges that can come with carrying and caring for a child.

Transcripts were analyzed in the language of the interview. To analyze the data, ZC and AB first reviewed all transcripts for accuracy to familiarize themselves with the data. During this process, both authors kept note of patterns in the data that, along with the interview guide, helped inform the development of a codebook. The final codebook included inductive and deductive codes. To guide coding, ZC and AB first coded the same transcript independently and then met with SA to review code placement and discuss areas of similar and different coding. Aligned with the goals of reflexive TA, our goal with this process was not to achieve coder agreement, but rather to examine and discuss the ways in which ZC and AB applied codes to the data to develop a deeper and richer analysis informed by both authors’ experiences and values. Both authors then continued to code independently, meeting regularly to discuss the coding process and make necessary revisions to the codebook based on the data. After completing coding, ZC and AB reviewed all coded data as a second step of familiarization and met with SA to begin identifying themes. First, the three authors utilized a virtual Whiteboard to facilitate a visual representation of codes, creating virtual sticky notes for each code and placing them on the virtual Whiteboard. We then examined the codes that appeared to have similar meanings together and arranged them into clusters. Next, we examined the codes within each cluster and identified a core concept for the cluster, which became a preliminary theme. We did this for all code clusters until we arrived at a preliminary list of themes. Next, AB checked preliminary themes against coded data and transcripts and found that all were supported by the data. ZC, AB, and SA then met again to refine the preliminary list, noting that two themes were capturing similar patterns of meaning in the data, which they condensed into one theme with a subtheme, ultimately settling on four overall themes. During the last phase of analysis, we further refined themes, adding a new subtheme, and condensing two main themes to develop our final results. Throughout the results, we present quotes from participants with identifiers for age, race/ethnicity, and whether they gave birth during or after the COVID-19 pandemic.

## Results

A total of 19 individuals participated in the qualitative study (Table 1). The sample was split nearly evenly between Black (*n* = 9) and Latina (*n* = 10) participants. Five participants primarily spoke Spanish. Eight gave birth before the onset of the COVID-19 pandemic (March 16, 2020), while the remaining 11 participants gave birth during the pandemic.

**Table 1:** Sample characteristics (*N* = 19)

	Births before COVID-19(*n*= 8)	Births during COVID-19(*n*= 11)
	*n*(%)	*N*(%)
Race/ethnicity		
Black	4 (50)	5 (45.5)
Hispanic/Latina	4 (50)	5 (45.5)
Both	0 (0)	1 (9.1)
Age at enrollment		
18–24	2 (25)	0 (0)
25–34	5 (62.5)	5 (45.5)
35 +	1 (12.5)	6 (54.5)
Education at first survey		
Some high school	1 (12.5)	2 (18.1)
Graduated high school or obtained GED	2 (25)	0 (0)
Some university, college, or technical	2 (25)	2 (18.1)
Completed technical school	1 (12.5)	3 (27.2)
Completed university or college	2 (25)	3 (27.2)
Prefer not to answer	0 (0)	1 (9.1)
Insurance status at first survey		
Private	2 (25)	6 (54.5)
MediCal	6 (75)	4 (36.3)
Don’t know	0 (0)	1 (9.1)
Number of kids at first survey		
0	3 (37.5)	2 (18.1)
1	1 (12.5)	2 (18.1)
2	0 (0)	3 (27.2)
3 +	1 (12.5)	3 (27.2)
Missing	3 (37.5)	1 (9.1)
Childhood discrimination (ever)^1^		
Rarely or never	3 (37.5)	5 (45.4)
Some of the time	3 (37.5)	1 (9.1)
Most of the time	2 (25)	4 (36.3)
Don’t know	0 (0)	1 (9.1)
Medical discrimination situations (ever)^2^		
None	5 (62.5)	6 (54.5)
1 to 3	1 (12.5)	0 (0)
4 to 7	2 (25)	5 (45.5)
Missing		
Number of situations with discrimination (ever)^1^		
None	2 (25)	1 (9.1)
1 to 3	3 (37.5)	0 (0)
4 to 7	1 (12.5)	4 (36.3)
8 +	0 (0)	1 (9.1)
Missing	2 (25)	5 (45.5)

1. Nancy Krieger and others, ‘Experiences of Discrimination: Validity and Reliability of a Self-Report Measure for Population Health Research on Racism and Health’, Social Science & Medicine, 61.7 (2005), 1576–96 <https://doi.org/10.1016/j.socscimed.2005.03.006>. 2. Peek, M. E., Nunez-Smith, M., Drum, M., & Lewis, T. T. (2011). Adapting the Everyday Discrimination Scale to Medical Settings: Reliability and Validity Testing in a Sample of African American Patients. Ethnicity & Disease, 21(4), 502–509.

In our analysis, we identified four themes relating to the impact of the COVID-19 pandemic on participants’ pregnancy, birth, and postpartum experiences: (1) the burden of becoming a mother and parenting during the COVID-19 pandemic; (2) the complexity of COVID-19 support and medical care delivery; (3) making the most of shelter-in-place; and (4) nurturing resilience and support during COVID-19.

### Theme 1: The Burden of Becoming a Mother and Parenting During the COVID-19 Pandemic

The 11 participants who were pregnant during the pandemic described the ramifications of the COVID-19 pandemic. Due to the concern of contracting COVID-19, participants were unable to access their support networks:

“Well, I don’t really feel comfortable being around people anymore. I haven’t hung out with my friends in a while, which really sucks. We used to hang out a lot, but I just don’t feel comfortable with people coming over, or going places, or if someone does come over, I just want to leave.”

−26-year-old, Latina woman, during COVID.

Being unable to access their support networks meant participants were unable to celebrate their pregnancy with loved ones:

*“*I mean, It was hard…Meaning you didn’t get to take the maternity photos. You didn’t get to take the-- you had the baby shower because it was when it was my time-- cause I delivered in May. And so, when it was my time to do all those things, it was like right in the height of it and nobody was doing nothing. So, we was like-- we didn’t have any of those things.”

- 32-year-old, Black woman, during COVID.

This theme uncovered challenges shared by participants whose older children transition from in-person to virtual school. Those participants who gave birth prior to COVID-19 were confronted with new parenting challenges. Many participants shared their frustrations with virtual school while raising their young babies and ensuring their older children were engaged in school.

“Ay, pues la verdad es muy estresante, la verdad. Porque, ay, no, imagínese. No es lo mismo que vayan a-- Sí está mejor que esté en la escuela ahorita por la-- que esté aquí en la casa ahorita por la pandemia. Para mí es mejor que esté en la casa, pero es muy estresante porque tengo que estar con la niña, tengo que estar con el niño. Ya me llama uno para allá y otro para acá. Entonces, no puedo estar con los 2. Sí es muy estresante.”

“Oh, well, the truth is very stressful, really. Because, oh no, imagine. It’s not the same as going to-- Yes, it’s better that I’m at school right now because of the-- that I’m here at home right now because of the pandemic. For me it’s better that he’s at home, but it’s very stressful because I have to be with the girl, I have to be with the boy. One calls me over there and another over here. So, I can’t be with both of them. Yes, it’s very stressful.”

−34-year-old Latina woman, prior to COVID.

A 34-year-old Latina participant who gave birth prior to COVID-19 decided to quit her full-time job to ensure her daughter adapted well to virtual school.

COVID-19 created a financial hardship to most of the participants. For participants who were not eligible for COVID-19 financial assistance or pregnancy disability leave due to their immigration status or not meeting state disability insurance criteria the financial constraints were further exacerbated. These participants relied on their savings, other social services, or their partners’ income, who often worked as essential workers and, as a result, had a heightened risk of COVID-19 infection for themselves and their families. One participant described how her financial challenges began with the loss of one of her part-time jobs. The loss of income impacted her ability to support her children and parents, who lived in her country of origin. On top of her financial constraints, she acquired COVID-19 during her pregnancy. Infected with COVID-19, she had to inject herself with blood thinner medication:

“Para mí ha sido muy complicado, porque el trabajo que yo tenía era de limpieza de casa, que era part time, pero me cancelaron todas las casa, porque yo tengo que mandarle dinero a mis hijos y a mi papá. Y sólo me quedé con el trabajo de la tienda. Ese es uno. La otra, que a mí me dio el COVID. Entonces, era doloroso inyectarme la, ¿cómo se llama ésta? Anticoagulante en la panza. Y lo sentía muy cansado para mí, muy agotador por el dolor. Esas fueron las gestiones y otra de las cosas, que como le digo, que nos bajaron las horas de trabajo a mi pareja y a mí.”

“For me, it has been really complicated, because the job I had was as a housecleaner, and it was part-time, but my clients canceled, and I have to send money to my children and my dad. And I only had the job at the store. That’s one. And another thing, I got COVID. So then it was painful for me to inject the, what do you call it? Anticoagulant injections in my stomach. And I found it very tiring, very exhausting because of the pain. Those were the challenges and other things, like I mentioned before, they cut our working hours for me and my partner.”

−37-year-old, Latina woman, during COVID.

Most of the participants noted that their financial security dwindled as a result of the pandemic, especially as their household went from having two to one working adult. COVID-19 social and health inequalities were exacerbated for participants considered essential workers such as healthcare, restaurants, cleaning services, education, and transportation workers. Some participants felt forced to leave unsafe employment, like one mother of four who quit her hospital job as a lactation consultant during her pregnancy due to improper COVID-19 precautions implemented by her employer.

“And so, once COVID happened, and I didn’t feel safe at work. I had to take myself out.

I’m not going to be in a position that’s going to jeopardize my health or my baby in any way. I requested a modification of duty to not have direct patient contact because initially, at the beginning of the pandemic, we did not have adequate PPE [personal protective equipment]. So, I didn’t feel safe. And when they refused to accommodate me, I decided to take leave-- It impacted me financially. It had an impact on my leave after having the baby, on my maternity leave. But I really wasn’t given any other option.”

−39-year-old Black woman, during COVID.

Another participant who acquired COVID-19 while working as a transit operator during her pregnancy shared her experience navigating COVID-19 safety guidance from her employer that she felt was improper and unjust:

“[I’m] trying to just be as informed as possible, talk to my support system, doing mindfulness you know, when things start to feel overwhelming, just trying to quiet the mind. And that’s all I really could do. It still was very difficult though. Especially in the job I was in, they did not care about COVID, didn’t care if you had PPE, didn’t care that I was pregnant, and there was a person with COVID on my vehicle. It was crazy.”

−37-year-old Black and Latina woman, during COVID.

Despite federal and state pandemic financial assistance in the form of stimulus payments, participants still struggled financially and sought additional assistance via community support in the form of food from local schools while others received support from pregnancy groups to increase their financial stability.

Furthermore, the isolation created by COVID-19 contributed to postpartum depression symptoms: more than half of participants who gave birth during COVID-19 reported symptoms of postpartum depression during shelter-in-place. Participants described intense irritability, anxiety, sadness, and difficulty cultivating confidence as a mother. Postpartum depression particularly impacted those with limited community and resource support. Notably, those who described the most significant challenges due to COVID-19 were single mothers who did not have a strong social support system. One single Black mother described the mental health challenges they experienced during the pandemic:

“For sure everyone can attest [the pandemic] has been crazy for everyone, but especially for me being a single mother and already experiencing postpartum depression just after having my child. And [my child] was roughly about five or six months when COVID hit. And that’s when I really became a single mother because I did have some help from my child’s father. But a month before COVID hit, that changed. And then when COVID hit, it just really took its toll on me.”

−33-year-old Black woman, during COVID.

As a single mother, this participant also found it difficult to access mental health services because they had no one to watch their daughter:

“Yeah. I did go to the hospital prior to COVID because I have epilepsy and I had a seizure while at home holding my daughter. So that also played a part in my anxiety. And then I also had a seizure during COVID, and I was home alone, but I was really scared to go to the hospital because of COVID, and also because being a single mother, I didn’t have that support of-- I’m pretty sure if I had reached out to somebody, they would have took her, but it would have just been a major inconvenience for me because of having the epilepsy, and I don’t try then relying on other people’s time to do things for me. So, I’ve had to learn to be self-sufficient and really not ask people for too much.”

−33-year-old Black woman, during COVID.

### Theme 2: The Complexity of COVID-19 Support and Medical Care Delivery

All participants who gave birth during the COVID-19 pandemic shared how their childbirth experiences and the level of support they received were altered due to ever-changing hospital policies as a result of the pandemic. These participants noted how they were restricted to one support person during and after birth, often opting for the baby’s father, despite wanting additional close family, friends, and, for some, their doulas present. One participant shared the increased need to have multiple support people to help ease emotions after a traumatic birth:

“[The pandemic] just made it hard. I think it would have been fine if I didn’t have such a traumatic birth, with my son being separated [from me]. I would have been OK, but because I had such a traumatic birth, it would have been nice to have one more person. My mom would have been the perfect person to hold me, calm me through it, and tell me things would be OK.”

−33-year-old Black woman, during COVID.

However, some people saw the silver lining of the COVID-19 support person restriction. For a 28-year-old Latina participant, she was able to frame this new policy with a positive perspective, expressing “Itkind of made it more intimate, just because it was just my boyfriend and myself there.So,it was nice, in a way, just being able to rely on each other and seeing him being so helpful.” This quote illustrates agency and resiliency by the participant asserting control over a precarious situation through valuing intimacy and a sense of mutual reliance between her and their partner. Although COVID-19 impacted extended labor support, many participants described how their medical team and labor support person found creative ways to recreate that essential care. One participant described the supportive care she received during her scheduled cesarean at the start of the COVID-19 pandemic:

“It was great. My partner was there with me. My whole team was just phenomenal, from the anesthesiologist to the supporting physician, they were just amazing. And we were listening to Beyonce while they were doing the surgery. So, it was great. The surgery went off really successfully.”

−35-year-old Black woman, during COVID.

One 37-year-old Black participant who gave birth during the COVID-19 described how the decision to have a homebirth allowed her to circumvent strict hospital policies and to be surrounded by family: “It worked out perfectly because I had kind of imagined that they’d all be around, that they’d be like here in the yard or something. I had this idea in my head that they would all be cheering me on.” This participant would not have been able to have her support circle with her had she given birth in a hospital setting. Having her family present for the birth and immediately after led her to feel “good” and “taken care of.”

In addition to labor support restrictions, this theme extended to COVID-19 medical care delivery modifications. All of the participants experience the transition from in-person to telehealth medical appointments. Some participants found telehealth appointments to suit their lives. A 28-year-old Latina participant found the telehealth appointments convenient since they did not have to leave their house, especially with a newborn. Others had concerns about the quality of telehealth appointments. A 34-year-old Latina participant who delivered prior to COVID-19 shared their preference for in-person appointments because the provider can perform a physical exam and ask necessary questions to properly make a diagnosis. Similarly, a participant shared the same concern, but found resistance from her baby’s pediatrician when she requested an in-person appointment:

I said well, aren’t you concerned about my baby, like, I said well, aren’t you concerned about my baby, like, should you be making sure he’s growing appropriately, you know what I mean? And he was like what number of baby is this for you? And I was like number 4, and then he was like you’re the experienced mom you know if your baby is doing well or not, and I’m like, what?

- 36-year-old Black woman.

#### Theme 3: Making the Most of Shelter-in-place

Despite the hardships brought on by the COVID-19 pandemic, the associated shelter-in-place orders afforded some participants a chance to slow down and spend more time with their families. Notably, nearly all participants who were pregnant during the COVID-19 pandemic described overall positive pregnancy experiences, even amidst unprecedented changes due to the pandemic. While there were financial constraints and new challenges, these participants attributed their ability to enjoy their pregnancy to their immediate family.

Some participants emphasized the positive impact of stay-at-home orders on their pregnancy and postpartum experience, especially for those who transitioned to work from home. These participants appreciated the extra quality time they had to rest and spend with family. One participant who gave birth before COVID-19 emphasized the joy they found in being able to be more present in her baby’s life due to a more flexible hybrid working schedule:

“When COVID shut everything down, it gave me a chance to take a breath and reorganize. And then I was able to spend more time with my kids, and help them with their schoolwork… I was so happy, like, oh, I don’t have to get up every day, get out the bed, get dressed, like it was awesome. And then I’m able to still work from home partially, so I know like pre-COVID that would not have been an option. They’re like, ‘OK, bring your butt back to work,’ but now I get to work two days in the office, and three days from home, which I’m happy about because I get to still see my baby grow up.”

−36-year-old Black woman, during COVID.

Another benefit of the extended time COVID-19 shelter-in-place provided was the ability to continue breastfeeding. As one participant described:

“Although the pandemic was crazy and totally screwed up a lot of the plans, it was really comforting to have this much quality time with my son. And I can only imagine how much more stressed I would have been as far as continuing to breastfeed him once I went back to work. So, this working from home has kind of been a saving grace. So yeah, it’s been a blessing.”

- 33-year-old Black woman, during COVID.

Prior to COVID-19, a 34-year-old Latina participant described compromising breastfeeding when returning to work.

“Pues como yo limpio casas, so, cuando podía me llevaba a la bebé. Y cuando no, pues se la dejaba una amiga, por eso era de que por eso le quería dar fórmula y pecho. Para que cuando no estuviera conmigo, pues pudiera comer, ¿verdad?”

“Well, since I cleaned the houses, so, when I could, I would take the baby with me. And when not, well, a friend left her, that’s why she wanted to give him formula and breast. So that when he wasn’t with me, he could eat, right?".

#### Theme 4: Nurturing Resilience and Support During COVID-19

Many participants described developing coping strategies and seeking support to navigate their pregnancies and parenting during the COVID-19 pandemic. Notably, a majority of participants noted how relying on their own resilience or their community support helped them ensure healthy pregnancies and parenting practices once the pandemic hit. One Black mother described her own coping strategy for navigating COVID-19 stressors during her pregnancy as managing her emotional response to the news:

“It’s nothing we’ve ever gone through before. So, it was a lot to try to absorb and take in and not feel anxious about. You’re hearing so much from around the world of people dying. It became a very intense, stressful situation. For me, it was important to try to stay grounded at all times and to not operate from a place of fear, even though it’s hard not to be fearful when you don’t know what’s gonna happen next. But it’s a lot when you’re trying to not manifest things and have it transferred to your baby.”

−37-year-old Black and Latina woman, during COVID.

Most participants expressed positive support by being connected with family and friends through phone or video calls. A 32-year-old Latina participant who delivered prior to COVID-19 explained she combated the feeling of isolation by video calls to her family in her country of origin. A 40-year-old Latina mother described how phone calls to her extended family mitigated her stressors while pregnant during COVID-19:

“Tenía a mi pareja, a mis cuñados, mis hermanos, aunque no viven aquí, pero siempre estaban ahí pendientes de que me cuidara, que no saliera cuando hablabamos por telefono.”

“I had my partner, my in-laws, my siblings. Even though they don’t live here, they were also there, making sure I took care of myself, that I didn’t go out, when we talked on the phone.”

Nearly all participants relied on support from their family or community to ensure a healthy pregnancy, including helpful advice and housing, financial, emotional, and childcare support.

At the individual level, participants implemented an array of health practices that supported their mental and physical health during COVID-19, which included following a healthy diet, engaging in physical activity, utilizing pregnancy mobile applications for pregnancy education, and practicing self-care in the form of going on walks, distraction, and breathing exercises. For example, one participant mitigated COVID-19 challenges by being in nature at the time of shelter-in-place orders during her pregnancy:

“Yeah, it was really perfect timing because where we previously lived was still a good.

area, but it was a little more urban, so there was a lot more congestion. And I probably wouldn’t have felt as comfortable going for walks just because of the amount of people I would probably encounter. Whereas where I’m living now it’s so quiet and peaceful, and yeah, we can go a while without seeing another person. The houses are spaced out a lot more, and there’s a huge park right outside where there are trails and stuff. So, there’s just a lot more opportunity to still be able to socially distance and be safe while getting exercise or just being in nature.”

−33-year-old Black woman, during COVID.

Two Black participants who were pregnant during the COVID-19 pandemic participated in virtual group prenatal care. The prenatal group was offered through their private midwifery care. Even though the virtual setting was not preferred, one of the 37-year-old Black woman appreciated the kinship developed with other pregnant people: “It was great. It really wasn’t likeclasses;it was more like just a moms’ group more than anything. But it was great. And we still talk now, the group.”

The rest of the participants who were unable to receive prenatal group support instead turned to their immediate family members, including partners, for pregnancy support. Some participants noted how their level of support increased during the pandemic, especially for those whose partners were privileged to work remotely or whose immediate family members lived nearby or in the same house. Participants embraced meditation practices, gardening, and contact with loved ones through Facetime and walks. These practices became a way for participants to find solace during their pregnancy and postpartum periods in the midst of a pandemic. Even with the adaptivity of the participants, medical and social support was essential for their empowerment through their pregnancy journey. As one 37-year-old-Black woman said:

“I think [healthy pregnancy is] just community, just having that support system that you can talk to people that you care about that also obviously care for you, that they show that they care for you, or are excited for you. Having that community and support.”

## Discussion

The COVID-19 pandemic upended daily life for all, bringing forth new challenges while magnifying existing ones. Particularly, the pandemic exacerbated structural inequities for Black and Latino families who experienced disproportionately high rates of COVID-19 infection and related mortality [23]. Furthermore, the pandemic complicated two especially vulnerable life phases: pregnancy and parenting [24–26], by limiting resources and support. In our study, participants recounted the difficulties they faced at home, work, and in hospital settings in the wake of the COVID-19 pandemic as they navigated pregnancy and postpartum parenting. Notably, while some of these difficulties were new, like having to take extra measures to stay safe from infection during pregnancy and being cut off from much-needed support circles due to isolating shelter-in-place orders, other struggles were all too familiar and experienced at an elevated level, such as financial instability. Nearly all participants in our study described having to navigate these two challenges during the pandemic, a finding in line with other studies documenting the impact of COVID-19 on parents [24, 27]. COVID-19 identified the gaps in health and social structures for vulnerable populations as mentioned above. At the same time, it also presented an opportunity to assess potential solutions to improve pregnancy and parenting outcomes to be applied post-pandemic. Our study’s goal is to showcase the complexities of pregnancy and parenting during COVID-19 through the lens of Black and Latina women.

During the early days of the pandemic, high-quality medical care was more important than ever due to the restriction of having extra support persons during hospital births. In our study, participants described disappointment and altered birth experiences from not being able to have more than one support person while birthing in hospital settings, specifically for those who had birth complications. These findings support recent research on the negative impact of COVID-19 on pregnancy and birth, particularly for those who gave birth in a hospital setting [28, 29]. Notably, some participants recounted how the level of care they received from their medical teams improved the quality of the COVID-19 medical constrictions, especially those who had access to midwives and hospital-based doulas. These findings highlight the positive impact a midwife or doula can have on their clients’ birthing experiences and support existing literature regarding the benefits of doula and midwifery care, particularly in Black and other BIPOC communities [29–33]. These findings can be used to advocate for increased access to this type of maternity care, particularly for Black and Latino birthing people, who often report receiving poor-quality maternity care [29, 34, 35]. Additionally, hospital management teams should consider the support needed to ensure a positive birth experience among all of their clients before implementing system-wide policies that can ultimately affect maternity care.

For some in our sample, the COVID-19 pandemic exposed significant needs regarding paid parental leave. Notably, the pandemic had a positive impact on those whose employers offered remote work or who had partners who were able to financially sustain the family. Having extra time at home allowed participants to relax, avoid outside stressors during pregnancy, and bond with their new babies and other children. Additionally, this privilege provided a sense of safety by reducing exposure to COVID-19 for this group of participants. Conversely, participants without the option of remote work, maternity leave, or prior financial instability reported an increase in financial stressors during the onset of the pandemic and COVID-19 exposure risks to themselves and their families. Additionally, some of these participants were diagnosed with postpartum depression, which made their transition to parenthood more difficult. Thayer and Glider (2020) validated similar findings with their study showing the association of financial stress caused by the COVID-19 pandemic their participants were twice the likelihood of having clinically significant depression [36]. This underscores the crucial need for financial stability following childbirth. In light of these findings, efforts should be made to expand access to parental leave benefits for Black and Latino parents, who often have unmet needs and, along with their infants, stand to benefit the most from this resource [37–39]. Furthermore, expanding the coverage of parental leave benefits should be a priority, given the ample evidence on the economic, health, and behavioral benefits of paid, extended parental leave [37, 40, 41]. Currently, the United States is the only high-resourced country that does not federally mandate a universal paid parental leave benefit.

This study gathered timely, important data on the impact of COVID-19 on Black and Latina women’s pregnancy, birth, and postpartum parenting experiences, adding to the growing literature base on the impact of the pandemic. By collecting qualitative data from Black and Latina women, we were able to gain a deeper understanding of how the pandemic compounded inequities in maternal health among these groups. The effects of the COVID-19 pandemic exacerbated financial instability during pregnancy and postpartum, cutting participants off from much-needed social and material support, creating a sense of isolation during hospital births, and, for those working outside the home, increased stress due to fears of COVID-19 infection. This increased stress and lack of support for coping intersected with the many hardships our participants, like many other Black and Latina women, faced before the pandemic, like financial and employment instability, and uniquely positioned them at an even higher risk for adverse maternal health and birth outcomes than they already experienced prior to the pandemic.

### Limitations

Limitations of the study include sampling from a singular geographic location, as well as the geographical context, which is characterized by high levels of structural instability and increased need for support.

### Conclusions

This analysis contributes to the existing scholarship on the impacts of the COVID-19 pandemic on birthing experiences, specifically among Black and Latina women and birthing people. Specifically, our study highlighted the important need for increased structural support for pregnant people and families, needs that were exacerbated by the pandemic. Our findings lend support to ongoing efforts advocating for the expansion of parental leave policies in the USA, particularly expanding paid coverage universally. Furthermore, our findings can be used to advocate for increased access to varied avenues of maternal care, including midwifery and doula care. Mitigating the effects of COVID-19, which disproportionately affect Black and Latino families and is still a relevant concern, is vital to avoiding compounding maternal health disparities in these communities.

## Data Availability

Data are available from the study’s Primary Investigators (BCB and AMG) via formal request. Requests are reviewed by the SOLARS Community Accountability Council, who also make the final decision regarding data sharing, to ensure that the proposed use of the data is aligned with the mission and principles of the group. Please reach out to the corresponding author if you wish to make a data request.
